# Patients Referred for Bleeding Symptoms of Unknown Cause: Does Evaluation of Thrombin Generation Contribute to Diagnosis?

**DOI:** 10.4084/MJHID.2016.014

**Published:** 2016-02-10

**Authors:** Elena Holm, Eva Zetterberg, Susanna Lövdahl, Erik Berntorp

**Affiliations:** Centre for Thrombosis and Haemostasis, Lund University, Skåne University Hospital, Malmö, Sweden

## Abstract

**Introduction:**

Patients with mild to moderate bleeding symptoms referred for coagulation investigation sometimes never receive a definitive diagnosis. Bleed assessment tools have been developed and validated to assess the severity of symptoms. Global coagulation assays, e.g., the thrombin generation test (thrombogram) have a potential to identify hemostatic defects that are not detected in specific assays.

**Material and Methods:**

One hundred and eighty-five patients referred to our centre because of bleeding symptoms were evaluated using the bleeding assessment tool (BAT) described by Tosetto and colleagues in 2006. Blood samples were investigated for thrombin generation (TG) capacity (Technoclone), in platelet poor (PPP) plasma, and specific clotting factors, i.e., von Willebrand factor, factor VIII and IX, as well as INR, APTT, platelet count, and platelet adhesion.

**Results:**

Of the 185 patients, five women were diagnosed with mild von Willebrand disease and one male with mild hemophilia A. The remaining 179 subjects (76% females and 24% males with average ages of 33 and 28 years, respectively) were evaluated further. In the total cohort and among women, peak TG, and lag time correlated with bleeding score (*p*=0.01 and *p*=0.04, respectively with correlation coefficients). No such correlations were found among males.

**Discussion and Conclusion:**

Although our study showed some correlation between TG and bleeding score, results are generally consistent with a previous report which failed to demonstrate the value of TG measurement in a similar setting. In conclusion, the complexity of the mechanisms underlying clinical bleeding complicates the ability to use TG tests as reliable predictors of bleeding. Mild congenital bleeding disorders, especially VWD, should be specifically screened for in patients with mild/moderate symptoms.

## Introduction

There are many patients with mild to moderate bleeding symptoms in whom no underlying coagulation defect can be found, despite thorough evaluation for clotting factor deficiencies, von Willebrand disease (VWD) and platelet function disorders. Typically, these patients are referred because of frequent bleeding episodes such as nose bleeds, heavy bleeding during menstruation, and bleeding during medical procedures. How to prevent haemorrhages during procedures, and offer targeted pharmacological treatment in the event of symptomatic bleeding in these patients, is currently left to the treating physician’s clinical judgement.

To assess the severity of a bleeding disorder, bleeding assessment tools (BATs) have been developed and validated. One BAT was described by Tosetto et al in 2006^1^ and was shortly thereafter introduced at our department as a routine tool in the check up of patients referred for bleeding symtoms of unknown cause. It was originally developed to discriminate VWD type 1, but has been used to evaluate other bleeding conditions as well.^2^ This BAT has been further modified and was in 2010 published as the ISTH BAT

Thrombin generation (TG) is a key process that determines the extent of a haemostatic plug or a thrombotic process. The inappropriate generation of thrombin may lead to haemorrhagic or thrombotic diseases. More than twenty years ago Hemker and colleagues^3^ introduced a method in which the amount of thrombin activity in plasma could be monitored continuously in platelet poor plasma (PPP) or platelet rich plasma (PRP). The method is currently referred to as thrombogram, previously thrombin generation curve, where dynamics of thrombin generation as well as the total amount of thrombin can be evaluated. Thrombin generation has been shown to be increased in patients with thrombotic disorders and decreased in patients with known clotting factor deficiencies such as haemophilia A and B.^4^,^5^

The aim of the present study was to assess whether thrombin generation parameters reflect the clinical phenotype in patients referred to a specialized centre because of bleeding symptoms, but where no diagnosis of clotting factor deficiency or platelet disorder could be determined.

## Materials and Methods

### Subjects

One hundred and eighty-five consecutive patients referred to the Centre for Thrombosis and Haemostasis in Malmö during the years 2008 through 2011 for bleeding symptoms of unknown cause were enrolled. Exclusion criteria were platelet count <80×10^9^/L, haemoglobin <10g/dL, known liver or kidney disease, use of medication such as aspirin, clopidogrel, non-steroidal anti-inflammatory drugs, antidepressants during the past seven days, anticoagulation therapy, and pregnancy as well as presence of a known clotting factor deficiency or platelet disorder.

Patients were included after signed informed consent and all study-related activities were performed according to the Helsinki declaration. The study was approved by the ethics committee, Lund University.

### Bleeding score

All patients were evaluated by the bleeding assessment tool described by Tosetto et al.^1^ and Bowman et al.^6^ The bleeding score (BS) obtained has been validated by Tosetto in adult patients with von Willebrand disease type 1, and a reference group without apparent bleeding symptoms.

The BS is calculated by summing the severity of all bleeding symptoms reported by a subject, and is graded according to an arbitrary scale. The grading of bleeding symptoms ranges from 0 (absence of abnormal haemorrhage) to 4 (blood transfusion and/or surgical intervention). In the case of no bleeding during two events of tooth extraction, surgery or delivery, one score is deducted from the sum. The BS has been used and validated for bleeding symptoms that were present at diagnosis before use of any prophylaxis. The cut off value described for persons without known bleeding disorders was set to <4.^1^,^6^

### Blood samples

Blood samples were collected by venepuncture using a 21-gauge needle into vacuum tubes (Vacutainer, Becton Dickinson, Plymouth, UK) containing 0.129 M sodium citrate without corn trypsin inhibitor (CTI), yielding a final concentration of 1:9 citrate/blood. The blood was centrifuged for 20 minutes at 1830 g, plasma transferred to a new tube and centrifuged for another 20 minutes, obtaining platelet poor plasma (PPP). The PPP was frozen to minus 80°C and thawed at 37°C just before performing the assay.

### Clotting factor and other laboratory analyses

To exclude the possibility of presence of a clotting factor deficiency or platelet disorder, the following routine assays were performed at the Coagulation Laboratory, Department of Clinical Chemistry: international normalized ratio (INR), activated partial thromboplastin time (APTT), platelet count, prothrombin time (PT), von Willebrand factor ristocetin cofactor activity (VWF:RCo)^7^ and von Willebrand factor Ag (VWF:Ag).^8^ Factor VIII was measured using a chromogenic assay (Coatest SP4, Chromogenix, Mölndal, Sweden) and FIX with a clotting based assay (Stago PTT Reagent). Platelet adhesion was measured using a modified Adeplat S test (Semmelweis, Milan, Italy) which is based on Hellem’s method.^9^ The test has a normal range of 16% – 34% with a coefficient of variation (CV) of 7.1% as tested in our laboratory. The intra-individual day-to-day variation has been tested in two healthy volunteers on six different occasions over a period of 4 years. The CV was found to be 8% and 13%, respectively.^10^

### Reagents

Human thrombin was obtained from Enzyme Research Lab (Dia-Service, Gothenburg, Sweden) and frozen (freeze dried) human plasma from the local plasma pool. All other reagents and buffer components were commercially available.

### Thrombin generation assay

The assay described by Varadi et al^11^ was used. Thrombin generation in citrated plasma (40uL) was triggered by a low concentration of TF/PL-complex phospholipid/tissue factor mix, (Rb containing 3,2 μM PCPS 80/20 (phosphatidyl-choline-phosphatidyl-serine) + 17,9 pM rTF. Technoclone Vienna, Austria), (10uL) in the presence of CaCl_2_ (15mM) and fluorogenic substrate Z-Gly-Gly-Arg-AMC (Bachem Ag, Bubendorf, Switzerland). Continuous fluorescence was measured on a FLx800 fluorescence luminescence reader (BioTek Instruments, Inc., Vermont, USA) and converted to fluorogenic units (RFU) by the kinetic program of the fluorometer. The rate of the increase of the RFU (RFU/min) was calculated at all-time points and converted to thrombin concentration by using a reference curve prepared from known concentrations of purified thrombin (Technothrombin TGA, Vienna, Austria.) From the resulting thrombin generation curve lag phase (time to thrombin to burst), peak thrombin and endogenous thrombin potential were calculated.

### Statistical analysis

Statistical analyses were performed using the Statistical Package for Social Sciences (SPSS version 20.0, SPSS; Chicago Illinois USA). A *p*-value <0.05 was considered to indicate statistical significance.

Correlations between variables were calculated with Spearman’s correlation. For comparison of the scores in the four age groups, the Kruskal Wallis test was used followed by a post hoc test using the Mann-Whitney U test with Bonferroni correction. Thus, a *p*-value < 0.008 was considered to indicate statistical significance.

## Results

Five unrelated women were diagnosed with mild VWD Type 1 and one male with mild haemophilia A. One of these women aged 21 was pregnant and had high levels of peak TG and bleeding score −1 (referred because of bruising). The other four women were 16, 41, 44 and 55 years old. They had bleeding score from the youngest to the oldest, 4, 7, 15 and 2. They all had low levels of peak TG. The diagnosis was set by a specialist in coagulations disorders based on clinical data and low values of VWF:RCo. The male with mild haemophilia A was 19 years old and had Factor VIII:C 0,14 kIU/L, bleeding score 3. Demographics of the 179 remaining subjects were as follows: women n=137 (77%) with a median age of 33 years (range 3–81) and males, n=42 (23%), median age 28 years (range 6–78). One hundred and twenty of the179 patients (64.5%) had a bleeding score <4 and 66 patients (33.5%) had a bleeding score ≥ 4. The main bleeding symptom for referral of each patient is given in Table 1. The most frequent symptoms encountered were menorrhagia, abnormal bleeding in connection with surgery, epistaxis and bruises. None of the patients was referred because of abnormal laboratory values. Four subjects were from one and the same family, without any common diagnosis after investigation. Otherwise subjects were not related.

**Figure 1:**
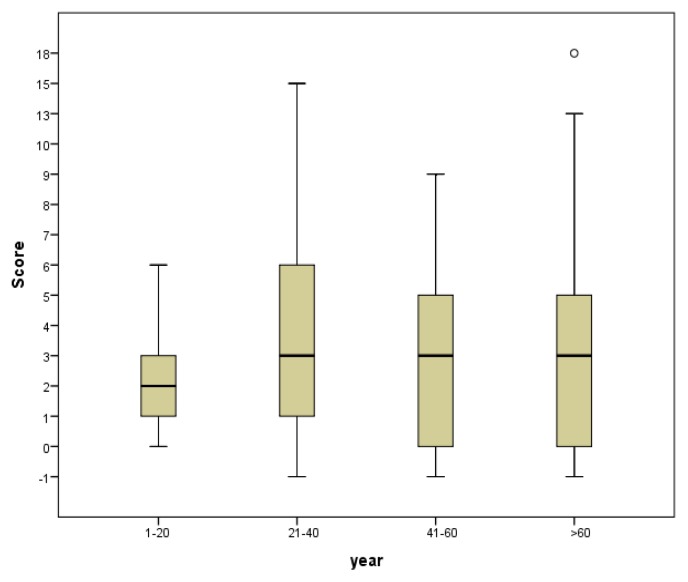
Bleeding score in different age cohorts

The entire cohort (n=179) was divided into four age groups (Figure 1). Group 0: 1–20 years (34.4%); Group 1: 21–40 years (33.3%); Group 2: 41–60 years (24.7%) and Group 3: >60 years (7.5%). The median bleeding score was lowest 2, in the youngest group (*p*=0.003) whereas the other groups had a score close to 3.

**Figure 2a and 2b:**
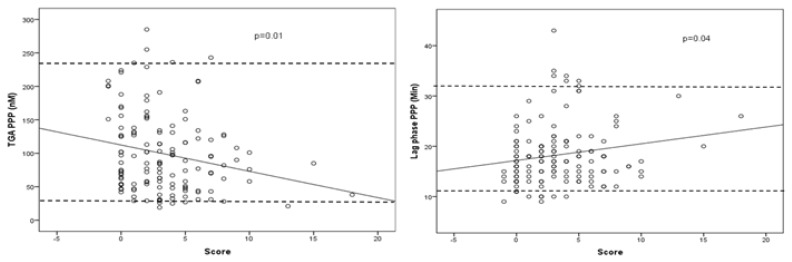
Correlation between bleeding score and peak thrombin generation (2a) and lag phase (2b) in female participants (n=137). Dotted lines indicate reference ranges for 11 hemostatically normal women (31–236 nM and 12–33 nM, respectively).

In the entire cohort, only peak thrombin significantly correlated with BS although the correlation coefficients were low. Among men, no such correlation was found. In contrast, peak TG and lag phase correlated significantly to bleeding score in women (Figure 2a and 2b) (*p*=0.01 and *p*=0.04, r=0.22 and r=0.18, respectively). There was no significant correlation between TG results and platelet adhesion (data not shown).

The most common laboratory deviation was low platelet adhesion value (<16%), as shown in Table 2. The 68 patients with low platelet adhesion values did not have higher bleeding scores than patients with normal platelet adhesion (data not shown).

## Discussion

Patients referred to our centre because of bleeding symptoms undergo clinical examination and a comprehensive medical history including administration of the Tosetto questionnaire which produces a bleeding score. This has been routine for many years and assures that a comprehensive history is taken, an important part in decision-making with respect to the direction of further investigations. Laboratory samples are routinely collected to exclude the most common coagulation disturbances. In many patients, abnormal values are never found, even in the presence of an obvious history of bleeding, and it is of importance to explore whether these cases have impaired haemostatic capacity as measured by thrombin generation capacity. The aim of our study, therefore, was to investigate whether there was a correlation between bleeding score and TGA parameters in patients at varying ages without known or diagnosed bleeding disorders. Correlations were seen in in the total cohort and in women, i.e., in PPP low values of peak thrombin and, also in women, long lag phase correlated with higher BS. These findings were not seen in males but this could be due to lack of statistical power, as men made up a much smaller proportion of the study cohort. The results indicate that some patients with bleeding tendencies have a lower capacity to produce thrombin upon haemostatic challenges, even when routine coagulation assays are shown to be normal. However the correlation coefficients were usually low which jeopardizes the practical value of the test in the clinical setting. Many, approximately 2/3, of our examined patients had low bleeding scores (<4) and most likely do not require further evaluation or treatment in connection with bleeding events or surgical procedures. Few patients with high BS had comparatively low peak thrombin generation. However, many patients with normal BS also had low thrombin generation capacity and the range of thrombin generation values in the BS group <4 was quite broad. A few patients (n=6) had abnormal values of APTT and/or PT but were not outliers in terms of TGA results. One of the patients had a bleeding score >4. None of the patients were considered to have a bleeding disorder. Of note is that five of the enrolled patients turned out to have VWD type 1 in mild form and one patient mild haemophilia A.

Our study has several strengths.

- Drawing and handling of blood samples and analysis of TG were done by the same staff during the entire period.
- Blood sampling was done in a stable situation when there was no bleeding.
- The intra-assay CV in the TGA test is below 10% at our laboratory.
- The BAT was administered by a small number of experienced physicians in a consistent manner. In addition, the number of patients evaluated is relatively high and represents a consecutively enrolled population referred to our centre.

Our study also has several limitations.

- The cross-sectional design where we could not evaluate weather TGA parameters was consistent with time. As our center is a referral center for large parts of the country additional sampling occasions were not feasible to perform.
- Mild platelet disorders cannot be ruled out using the platelet retention test which was the only routine test available during the entire study period. The experience from our laboratory is that the specificity and sensitivity of the platelet retention test for bleeding symptoms are rather low in subjects where no indication of plasma coagulation aberration can be revealed (unpublished data from a population-based prevalence study on bleeding disorders in young women).^12^ This is substantiated by the finding in the present study that 68 subjects with low platelet adhesion values did not have higher bleeding scores than those with normal platelet adhesion.
- Controls not referred for bleeding symptoms were not included.

One previous study^2^ compared TGA parameters in patients with mild to moderate bleeding complications with age and gender matched controls, and no significant differences were found. The findings in our study corroborate, to some extent, the previous report but also show a correlation between thrombin generation parameters analysed in PPP and bleeding scores in women. Given that these findings derive from a large group, it is not clear whether they are of significant value in the evaluation of an individual patient. In those with high BS, however, TGA could add value in the routine check up to discriminate between platelet function impairment and impairment of the plasma coagulation system, even if routine assays such as APTT and PT are normal.

The outcome of the study is not only dependent on the TG assay but also on the quality of the BS assessment. Several bleeding assessment tools (BATs) have been published and in 2010 ISTH/SSC proposed a standardized questionnaire developed from previously published tools^13^ with the intention to improve the sensitivity of BATs across all age groups. As our study started prior to publication of the ISTH/SSC-BAT we have not been able to include this tool in the calculations.

## Conclusions

The complexity of clinical bleeding – an interplay of coagulation and fibrinolysis, platelets and vessel walls, as well as life style – complicates the ability to identify thrombin generation, an apparent key parameter, as having major importance as a predictor of haemorrhagic tendency. Sensitive platelet function tests, usually not available as routine analyses, perhaps might disclose more abnormalities and explain mild bleeding symptoms. The study also show the importance of looking for mild congenital bleeding disorders such as VWD type 1 and mild hemophilia in patients referred with mild/moderate bleeding symptoms.

## Figures and Tables

**Table 1:** Description of primary bleeding type in each patient as cause for referral.

Bleeding	N (%)
Menorrhagia	49 (27.4)
Abnormal bleeding in connection with surgery	36 (20.1)
Epitaxis	34 (18.9)
Bruises	28 (15.6)
Postpartum bleeding	6 (3.4)
Extreme bleeding after tooth extraction	5 (2.8)
Hematuria	3 (1.7)
Gastrointestinal bleeding	3 (1.7)
Joint and muscle bleeding	2 (1.1)
Other	13 (7.3)
Total	179 (100)

**Table 2:** Laboratory results

Laboratory parameter	n	Median (range)	Normal range
APTT, s	179	31 (21–49)	26–33
FVIII level, kIU/L	179	1.06 (0.39–2.14)	0.50–2.00
FIX activity, kIU/L	25	0.94 (0.58–1.64)	0.70-1-30
Platelet count, ×10^9^/L	179	251 (106–449)	165–387 (female) 145–348 (male)
INR	179	1.0 (0.9–1.8)	<1.2
PT, s	179	12.11 (10–21)	10–13
Platelet adhesion, %	179	15.46 (3–30)	16–34
VWF:RCo kIU/L	179	0.90 (0.44–1.5)	0.52–1.58
VWF:Ag, kIU/L	30	0.76 (0.4–1.92)	0.60–2.73
